# Combined effects of landscape composition and heterogeneity on farmland avian diversity

**DOI:** 10.1002/ece3.2693

**Published:** 2017-01-25

**Authors:** Joana Santana, Luís Reino, Chris Stoate, Francisco Moreira, Paulo F. Ribeiro, José L. Santos, John T. Rotenberry, Pedro Beja

**Affiliations:** ^1^ CIBIOCentro de Investigação em Biodiversidade e Recursos Genéticos/InBIOUniversidade do PortoVairãoPortugal; ^2^ CEABNCIBIOCentro de Investigação em Biodiversidade e Recursos Genéticos/InBIOInstituto Superior de AgronomiaUniversidade de LisboaLisboaPortugal; ^3^ CIBIOCentro de Investigação em Biodiversidade e Recursos Genéticos/InBIOUniversidade de ÉvoraÉvoraPortugal; ^4^ Game & Wildlife Conservation TrustAllerton ProjectLoddingtonLeicsUK; ^5^ CEFCentro de Estudos FlorestaisInstituto Superior de AgronomiaUniversidade de LisboaLisboaPortugal; ^6^ Department of Ecology, Evolution, and BehaviorUniversity of MinnesotaSaint PaulMinnesotaUSA

**Keywords:** agriculture intensification, biodiversity conservation, bird species richness, compositional heterogeneity, configurational heterogeneity, landscape composition

## Abstract

Conserving biodiversity on farmland is an essential element of worldwide efforts for reversing the global biodiversity decline. Common approaches involve improving the natural component of the landscape by increasing the amount of natural and seminatural habitats (e.g., hedgerows, woodlots, and ponds) or improving the production component of the landscape by increasing the amount of biodiversity‐friendly crops. Because these approaches may negatively impact on economic output, it was suggested that an alternative might be to enhance the diversity (compositional heterogeneity) or the spatial complexity (configurational heterogeneity) of land cover types, without necessarily changing composition. Here, we develop a case study to evaluate these ideas, examining whether managing landscape composition or heterogeneity, or both, would be required to achieve conservation benefits on avian diversity in open Mediterranean farmland. We surveyed birds in farmland landscapes of southern Portugal, before (1995–1997) and after (2010–2012) the European Union's Common Agricultural Policy (CAP) reform of 2003, and related spatial and temporal variation in bird species richness to variables describing the composition, and the compositional and configurational heterogeneity, of the natural and production components of the landscape. We found that the composition of the production component had the strongest effects on avian diversity, with a particularly marked effect on the richness of farmland and steppe bird species. Composition of the natural component was also influential, mainly affecting the richness of woodland/shrubland species. Although there were some effects of compositional and configurational heterogeneity, these were much weaker and inconsistent than those of landscape composition. Overall, we suggest that conservation efforts in our area should focus primarily on the composition of the production component, by striving to maximize the prevalence of biodiversity‐friendly crops. This recommendation probably applies to other areas such as ours, where a range of species of conservation concern is strongly associated with crop habitats.

## 1 Introduction

Conserving biodiversity on farmland is essential for reversing the global biodiversity decline, but achieving this goal has been hindered by the pervasive intensification of agricultural land uses (Donald, Sanderson, Burfield, & Van Bommel, 2006; Krebs, Wilson, Bradbury, & Siriwardena, 1999; Sutcliffe et al., 2015). Changing landscape composition (i.e., the type and amount of different land cover types) by increasing land cover by natural or seminatural habitats preserved in agricultural landscapes (e.g., hedgerows, scrublands, riparian vegetation, woodlands, and ponds) might benefit biodiversity, as they provide key habitats for plants and animals (Ricketts, 2001; Wethered & Lawes, 2003), and they may act as corridors or stepping stones that facilitate dispersal among more natural areas (Fischer & Lindenmayer, 2002; Hinsley & Bellamy, 2000). However, significantly increasing the amount of natural habitats may be difficult or even impossible in many cases, because there is growing pressure for conservation on farmland to have minimal impacts on agricultural economic output (Fischer et al., 2008; Green, Cornell, Scharlemann, & Balmford, 2005; Tscharntke et al., 2012).

Meeting conservation objectives without increasing the amount of natural habitats might be achieved through changes in the crops produced, because different crop types have different structural characteristics and are associated with distinct agricultural practices that may strongly influence farmland biodiversity (Ribeiro, Santos, Santana, Reino, Beja, et al., 2016; Stoate et al., 2009). In northern Europe, for instance, sowing cereals in spring rather than in autumn increases nest sites for birds (Berg, Wretenberg, Żmihorski, Hiron, & Pärt, 2015; Chamberlain, Fuller, Garthwaite, & Impey, 2001), while producing late‐harvested hay rather than early‐harvested silage improves foraging habitats and increases avian nesting success (Butler, Boccaccio, Gregory, Vorisek, & Norris, 2010). Also, farmland plants, arthropods, and birds are benefited by annual crops and pastures with more heterogeneous and sparser swards (Wilson, Whittingham, & Bradbury, 2005). The production on former arable land of permanent crops such as olive orchards or energy crops such as willow short rotation coppice may also increase biodiversity, by attracting shrubland and woodland species to farmland (Rey, 2011; Sage, Cunningham, & Boatman, 2006). Despite these potential benefits, however, changing crop types on private land may be difficult, because this is conditional on complex farmers' decisions driven by a combination of agricultural policies, biophysical and socioeconomic constraints, and market demands (Ribeiro et al., 2014).

Given these difficulties, it was recently suggested that efforts should concentrate on managing landscape heterogeneity (i.e., the diversity and spatial pattern of land cover types), without necessarily changing landscape composition (Fahrig et al., 2011). These efforts may focus on either the natural (i.e., natural and seminatural habitats) or the production (i.e., different arable crops, grazed lands, orchards) components, or both, aiming to increase the compositional (i.e., richness or diversity of land cover types) or configurational heterogeneity (i.e., complexity in the spatial arrangement of land cover types, for example, diversity of patch sizes and shapes, and edge density), or both (Fahrig et al., 2011). This strategy seems sensible, because increasing the number of cover types may increase conditions for a larger number of species with contrasting ecological requirements, thus generating higher species richness (Pickett & Siriwardena, 2011; Stein, Gerstner, & Kreft, 2014). Likewise, high diversity of cover types may favor the persistence of species that use different habitats during their life cycle or throughout the year (Benton, Vickery, & Wilson, 2003; Chamberlain, Wilson, Browne, & Vickery, 1999). Increasing configurational heterogeneity may also be important, because it increases the length of ecotones and interspersion/juxtaposition of habitats, which are favorable for many species (Fahrig et al., 2011; Tryjanowski, 1999). These ideas based on landscape heterogeneity may thus provide a valuable framework to improve biodiversity conservation on farmland (Batáry, Báldi, Kleijn, & Tscharntke, 2010; Concepción et al., 2012), although its practical application in real landscapes would require further information on the relative importance of landscape composition versus heterogeneity, as well as on the relative role of the different heterogeneity components.

**Figure 1:**
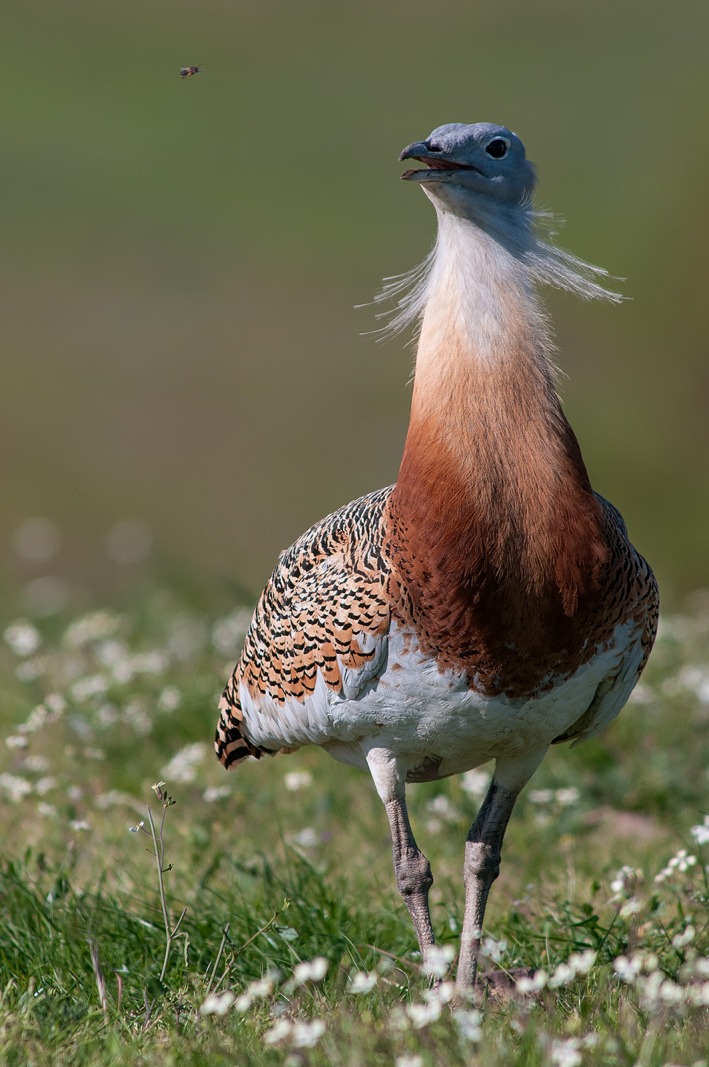
Great bustard (*Otis tarda*) breeding male in a grassland area within the Special Protection Area of Vila Fernando, Elvas, southern Portugal. Photograph by Luís Venâncio

Here, we address these issues evaluating how landscape composition and heterogeneity affect spatial and temporal variation in avian diversity in Mediterranean farmland landscapes of southern Portugal. We focused on an extensive farmland area included in a Special Protection Area created to protect steppe bird species (Figure 1) of conservation concern (Santana et al., 2014; and references therein) and on a neighboring farmland area dominated by intensive agricultural land uses (Ribeiro et al., 2014). The study covered periods before (1995–1997) and after (2010–2012) the European Union's Common Agricultural Policy (CAP) reform of 2003, thus encompassing major changes in agricultural land uses and practices (Ribeiro, Santos, Santana, Reino, Beja, et al., 2016; Ribeiro, Santos, Santana, Reino, Leitão, et al., 2016; Ribeiro et al., 2014), and in bird assemblages (Santana et al., 2014), in both study areas. Based on previous ecological studies on the bird species of this region (e.g., Delgado & Moreira, 2000, 2002; Reino et al., 2009, 2010), we tested the following expectations: (1) landscape composition of the natural component should be a strong driver of spatial and temporal variation in bird diversity, with a particularly strong positive effect of the amount of natural habitats on woodland and shrubland species; (2) landscape composition of the production component should also be influential, particularly for farmland and steppe bird species; (3) landscape compositional and configurational heterogeneity should add significantly to landscape composition in influencing bird diversity; and (4) landscape heterogeneity of the natural component should be most influential on woodland and shrubland species, while effects of the production component should be stronger on farmland and steppe birds. Results of our study were used to discuss the importance of considering landscape composition and heterogeneity of both the production and natural components when managing farmland landscapes for conservation, and how this importance may vary widely in relation to conservation objectives.

## 2 Materials and Methods

### 2.1 Study area

**Figure 2:**
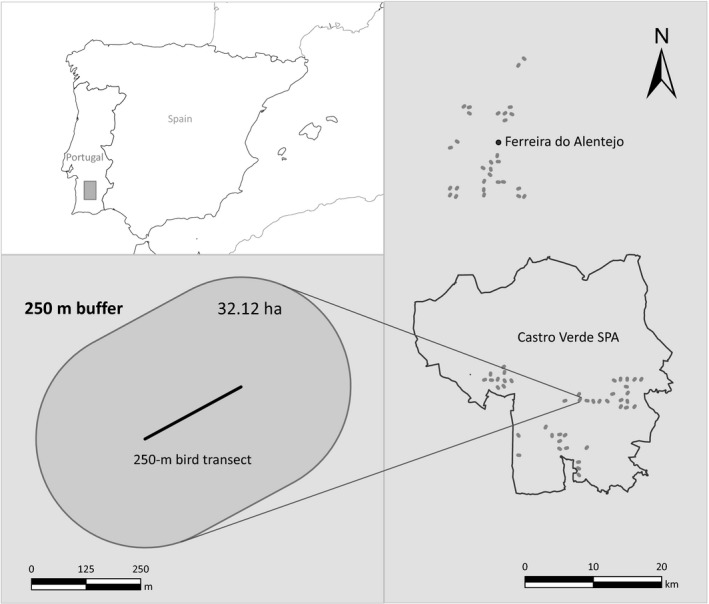
The study area in southern Portugal, showing its location in the Iberian Peninsula (upper left panel), the distribution of 73 250‐m bird sampling transects in relation to the Special Protection Area (SPA) of Castro Verde (right panel), and an example of a 250 m buffer around a transect where landscape composition and heterogeneity were characterized (lower left panel)

The study was conducted in a Mediterranean agricultural region of southern Portugal (Figure 2), within a low‐intensity farmland area included in the Special Protection Area (SPA) of Castro Verde (37° 41′ N, 8° 00′ W) and within the nearby (about 10 km) high‐intensity farmland area of Ferreira do Alentejo (38° 03′ N, 8° 06′ W). Before the CAP reform of 2003, agriculture in the low‐intensity area was dominated by the traditional rotation of rain‐fed cereals and fallows typically grazed by sheep, which provides habitat for a range of steppe bird species (Delgado & Moreira, 2000; Santana et al., 2014). Following the CAP reform, there were marked shifts from the traditional system toward the specialized production of either cattle or sheep, with declines in cereal and fallow land, and increases in permanent pastures (Ribeiro et al., 2014). Throughout the study period, this area benefited from significant conservation efforts, including agri‐environment schemes, legal restrictions to afforestation and land use intensification, and projects targeting steppe birds (Ribeiro et al., 2014; Santana et al., 2014). In contrast to Castro Verde, the high‐intensity area had irrigation infrastructures, better soils, and no constraints to crop conversion (Ribeiro et al., 2014). Before the CAP reform, agriculture in this area was dominated by intensive, annual irrigated crops, but thereafter there was a progressive shift to permanent crops (mainly olive groves; Ribeiro et al., 2014).

### 2.2 Study design

The study was based on the modeling of spatial and temporal variation in the species richness of breeding bird assemblages in relation to variables describing landscape composition and heterogeneity. Spatial variation was analyzed on two occasions, corresponding to periods before (1995–1997) and after (2010–2012) the CAP reform of 2003. Temporal variation was estimated from differences in richness between the two time periods. Sampling was based on a network of 250‐m transects set in 1995, which were initially designed to evaluate the effects of an agri‐environment scheme, with 46 transects set in the SPA of Castro Verde and 32 in the nearby area of Ferreira do Alentejo (Santana et al., 2014; Stoate, Borralho, & Araújo, 2000). Transects followed a random bearing, and they started at grid intersections of a 1‐km square grid overlaid on the study area, which were selected based on access constraints and the presence of agricultural land uses (Stoate et al., 2000). From the initial pool of 78 transects, we retained 73 that were surveyed in at least 2 years in each of the two time periods (mean counts per transect ± *SD*; min–max = 5.8 ± 0.4; 5–6). Landscape variables were estimated within 250‐m buffers (32.12 ha) of each transect (Figure 2).

### 2.3 Bird surveys

Birds were sampled three times per time period in each transect, corresponding to one sampling occasion per year and transect in 1995–1997 and 2010–2012. Sampling was conducted during the breeding season in April–May, which was deemed adequate to maximize the chances of detecting both resident and trans‐Saharan migratory species (Reino et al., 2009, 2010). Transects were walked in early morning and late afternoon, and all birds observed within 250 m were registered (Santana et al., 2014). Species richness was estimated from the total number of species registered per transect in either 1995–1997 or 2010–2012. Bird data were pooled per time period to increase species detectability and to minimize potential confounding effects resulting from year‐to‐year fluctuations in species occurrences unrelated to local habitat conditions, differences in observer skills, and the possibility of missing some species when sampling on a single sampling occasion per year. To test for differential landscape effects on different species groups, we computed both the total species richness and the richness of species categorized according to major habitat affinities (Table S1): (1) woodland birds—species dependent on woodlands and shrublands; (2) farmland birds—species associated with all farmland habitat types (e.g., arable fields, permanent crops, hedgerows); and (3) steppe birds—a subset of farmland species occurring only in open grassland habitats (Gil‐Tena, Saura, & Brotons, 2007; Reino et al., 2009, 2010; Santana et al., 2014). Aquatic birds were excluded because they were inadequately sampled by our approach. See Santana et al. (2014) for methodological details.

### 2.4 Landscape composition and heterogeneity

For each buffer around each transect, we prepared land cover maps for 1995–1997 and 2010–2012, using digital aerial photographs from 1995 (scale 1:40,000), and Bing Aerial images from October 2010 to July 2011 (http://mvexel.dev.openstreetmap.org/bing/), respectively. The minimum mapping unit was 50 m^2^, and we differentiated all land cover categories that could be readily identified in the photographs. Using a single land cover map for each 3‐year period was considered reasonable because bird data were also pooled for the same periods and because land cover categories were not expected to drastically change within each period. Mapping was refined with information from a governmental database of agricultural land uses at the parcel scale (details in Ribeiro et al., 2014), using data from 2000 and 2010 to represent crop types in 1995–1997 and 2010–2012, respectively. The 3‐ to 5‐year mismatch in the first period was considered reasonable, because it corresponded to a time of relative stability in agricultural land uses before the Common Agricultural Policy (CAP) reform of 2003 (Ribeiro et al., 2014). Therefore, no major annual variations in the production component were expected, particularly considering the broad land cover categories used. Furthermore, the information on agricultural land uses was cross‐checked with information from aerial photographs and the official land cover maps of Continental Portugal for 1990, further guaranteeing that no significant land use changes would be missed. Cartography for 2010–2012 was further refined using the official land cover maps of Continental Portugal for 2007.

Detailed land cover types in the preliminary map were categorized in 11 broad categories, which were defined to have management relevance (e.g., Ribeiro, Santos, Santana, Reino, Beja, et al., 2016; Ribeiro et al., 2014) and to reflect functionally important habitats for regional bird assemblages (Figure S1). Specifically, we considered categories reflecting the natural component of the landscape (woodlands, open woodlands, shrublands, streams, and water bodies), which were expected to be particularly important for different woodland and shrubland species, and categories reflecting the production component (annual dry crops and fallows, permanent pastures, annual irrigated crops, arable land with scattered trees, and permanent crops), which were expected to be particularly important for different farmland species (e.g., Delgado & Moreira, 2000; Moreira, 1999; Reino et al., 2009, 2010; Santana et al., 2014; Stoate et al., 2000). Landscape composition was then estimated as the proportional cover by each land cover category. The same categories were used to estimate variables describing the heterogeneity of both the natural and production components of the landscape. Following Fahrig et al. (2011), landscape compositional heterogeneity was described from the richness, diversity, and evenness of land cover categories, while landscape configurational heterogeneity was described from the largest patch index, mean patch size, edge density, and mean shape complexity (details in Table S2). Landscape metrics were estimated in a GIS using Fragstats 4.2 (McGarigal & Ene, 2013).

### 2.5 Statistical analysis

In each time period, we modeled spatial variation in species richness in relation to landscape variables using generalized linear models (GLM) with Poisson errors and log link (dispersion parameter close to 1, mean ± *SD* = 1.06 ± 0.38), while we used GLMs with Gaussian errors and identity link to model temporal variations in species richness. In temporal analyses, variations in species richness were measured by subtracting species richness of 1995–1997 from that of 2010–2012, while temporal variation in landscape variables was estimated likewise by subtracting the values of the first period from those of the second (e.g., Δ Edge density = Edge density [2010–2012] – Edge density [1995–1997]). Before analysis, landscape variables were transformed using the angular transformation for proportional data and the logarithmic transformation for continuous variables, thereby minimizing potential problems associated with the unit sum constraint and the undue influence of extreme values.

Model building procedures were based on the information theoretic approach with multimodel inference (Burnham & Anderson, 2002). First, we estimated for each dependent variable the relative importance of landscape composition, compositional heterogeneity, and configurational heterogeneity, of either the natural or the production components (Table 1), based on 63 a priori candidate models corresponding to all possible combinations of these six sets of variables (Table S3). Each set appeared in the same number of models (32), and each variable appeared in a model with every other variable. For all candidate models, we calculated model probabilities (Akaike weights, *w*_*i*_) based on Akaike information criterion corrected for small sample sizes (AICc). The importance of each set of variables was then calculated by the sum of the *w*_*i*_ (*w*_*i*_+*)* of the models where each variable set was present. Second, sets of variables with *w*_*i*_+ > 0.5 were carried over to a subsequent modeling step, where we built average models to evaluate the importance of each individual variable to explain variation in species richness. In this case, candidate models were built from all combinations of variables included in analysis.

**Table 1:** Summary statistics (mean ± standard error [*SE*]; minimum [Min], and maximum [Max]) of variables describing landscape composition and heterogeneity in 250 m buffers around 73 transects used to estimate bird species richness in 1995–1997 and 2010–2012, in southern Portugal

Landscapes variables	1995–1997		2010–2012		Temporal variation		Paired *t*‐test	
	Mean ± *SE*	Min, Max	Mean ± *SE*	Min, Max	Mean ± *SE*	Min, Max	*t*	*p*
**Natural component**								
**[1] Composition**								
Woodland	2.3 ± 1	0, 58.2	1.5 ± 0.5	0, 23.5	−0.8 ± 0.7	−47.4, 10.3	−0.84	.403
Open woodland	6.7 ± 2.1	0, 80	7.9 ± 2.4	0, 78.4	1.3 ± 1.4	−33.4, 54.6	0.74	.462
Shrubland	1.4 ± 0.3	0, 12.9	1.4 ± 0.4	0, 20.9	0 ± 0.2	−6.6, 10.2	−1.72	.091
Streams	1.1 ± 0.3	0, 15.2	1.1 ± 0.3	0, 15.2	0 ± 0.1	−2.5, 1.3	−0.28	.783
Water bodies	0.1 ± 0.0	0, 2	0.6 ± 0.2	0, 16.5	**0.5 ± 0.2**	**−0.1, 16.5**	***3.10***	***.003***
**[2] Compositional heterogeneity**								
Land cover richness	1.5 ± 0.1	0, 4	1.5 ± 0.1	0, 5	0.1 ± 0.1	−1, 2	0.75	.456
Land cover diversity	0.3 ± 0.0	0, 1.1	0.3 ± 0	0, 1.3	0 ± 0	−0.6, 0.6	−0.17	.863
Land cover evenness	0.3 ± 0.0	0, 1	0.3 ± 0	0, 1	0 ± 0	−0.9, 0.8	−0.18	.854
**[3] Configurational heterogeneity**								
Largest patch index	6.3 ± 1.8	0, 73.7	7.1 ± 1.9	0, 72.8	0.8 ± 0.8	−21.2, 49.8	1.07	.289
Patch size	0.6 ± 0.2	0, 11.1	0.7 ± 0.2	0, 11.2	0.1 ± 0.1	−1.3, 3.4	1.03	.304
Edge density	68.3 ± 10.1	0, 340.9	67.5 ± 10.8	0, 387.3	−0.8 ± 3.8	−127, 88.8	−0.08	.933
Shape complexity	2.1 ± 0.2	0, 7.5	2 ± 0.2	0, 6.9	0 ± 0.1	−4, 3.4	0.29	.770
**Production component**								
**[4] Composition**								
Arable land with scattered trees	4 ± 1.1	0, 59.3	2.4 ± 1	0, 59.4	**−1.6 ± 0.6**	**−34.5, 1.7**	**−*3.05***	*.003*
Annual dry crops	50.2 ± 3.8	0, 100	20.8 ± 3.3	0, 99.4	**−29.4 ± 4.4**	**−98.9, 72.7**	**−*6.82***	***<.001***
Permanent pastures	17.7 ± 3.4	0, 99.6	36.6 ± 4.6	0, 99.4	**18.9 ± 3.9**	**−51.2, 99.4**	***4.83***	***<.001***
Annual irrigated crops	14.6 ± 2.9	0, 95.6	8.8 ± 2.3	0, 87.6	**−5.7 ± 2.6**	**−95.6, 51.3**	**−*2.74***	***.008***
Permanent crops	1.6 ± 0.7	0, 47.8	18.2 ± 3.9	0, 100	**16.6 ± 3.8**	**−8.3, 100**	***4.30***	***<.001***
**[5] Compositional heterogeneity**								
Land cover richness	2.3 ± 0.1	1, 4	2.2 ± 0.1	1, 4	−0.1 ± 0.1	−2, 1	−1.16	.252
Land cover diversity	0.5 ± 0	0, 1.2	0.4 ± 0	0, 1.1	**−0.1 ± 0**	**−0.8, 0.7**	**−*2.61***	***.011***
Land cover evenness	0.6 ± 0	0, 1	0.4 ± 0	0, 1	**−0.1 ± 0.1**	**−1, 0.9**	**−*2.73***	***.008***
**[6] Configurational heterogeneity**								
Largest patch index	61.6 ± 3.1	5.2, 100	63.7 ± 3.2	9.5, 100	2.1 ± 2.2	−64.5, 48.1	1.24	.219
Patch size	10 ± 0.9	0.3, 32.1	10.1 ± 0.9	0.4, 32.1	0.1 ± 0.9	−23.1, 22.7	−0.02	.980
Edge density	90 ± 7.5	0, 346.6	82.6 ± 8.1	0, 366.4	−7.4 ± 4.6	−151.1, 144.7	−1.50	.138
Shape complexity	1.8 ± 0.1	1.2, 3.6	1.7 ± 0	1.1, 3.1	−0.1 ± 0	−1.4, 0.9	−1.46	.148

Temporal variation indicates differences between the second and the first period, and significant deviations from zero (*p *<* *.05; paired *t*‐test) are in bold. Variables are organized according to six sets [#] used in data analysis. Landscape composition variables are expressed in percentage cover (%) and are described in Figure S1. Description and units of heterogeneity variables are given in Table S2.

To assess the relative importance of variables and to build average models, we used the procedure of Cade (2015), which explicitly acknowledges that the independent variables were intercorrelated to greater or lesser degrees and that the statistical expression of the effects of one variable may change depending upon which other variables are included in any particular model (Cushman, Shirk, & Landguth, 2012; Herzog et al., 2006). Therefore, we computed model averaging for the partial standardized coefficients obtained by multiplying the unstandardized coefficient in the model by the partial standard deviation of the variable, which is a function of the standard deviation of the variable in the sample, the sample size, the number of variables in the model and the variance inflation factor of the variable (Cade, 2015). Then, we estimated the relative importance of each variable within each model as the ratio of its partial standardized regression coefficient (absolute value) to the largest partial standardized regression coefficient (absolute value) in the model (Cade, 2015). This approach examines the importance of each set of variables in the context of every other combination of variable sets, and the importance of each individual variable in the context of its contribution relative to other variables in a model, independently of the variable set (Cade, 2015).

To evaluate spatial autocorrelation problems that might produce biased model coefficients (Diniz‐Filho et al. 2008), we used spline correlogram plots with 95% pointwise confidence intervals calculated with 1,000 bootstrap resamples (Bjørnstad & Falck, 2001). We inspected correlograms for both the raw data and model residuals, to assess whether autocorrelation was effectively removed in the modeling process. We assumed that variable selection and parameter estimation were unbiased when there was no significant autocorrelation in model residuals (Diniz‐Filho et al. 2008, Rhodes, McAlpine, Zuur, Smith, & Ieno, 2009).

All analyses were performed using R 3.2.5 (R Core Team 2016). GLMs were performed using “glm” function in MASS package (Venables & Ripley, 2002), Akaike weights were calculated using “akaike.weights” function in qpcR (Spiess, 2014), model averaging was performed using “model.avg” and “partial.sd” functions in MuMIn (Bartoń, 2016), and spline correlograms were plotted using “spline.correlog” and “plot.spline.correlog” functions in ncf (Bjørnstad & Falck, 2001).

## 3 Results

### 3.1 Overall patterns

**Figure 3:**
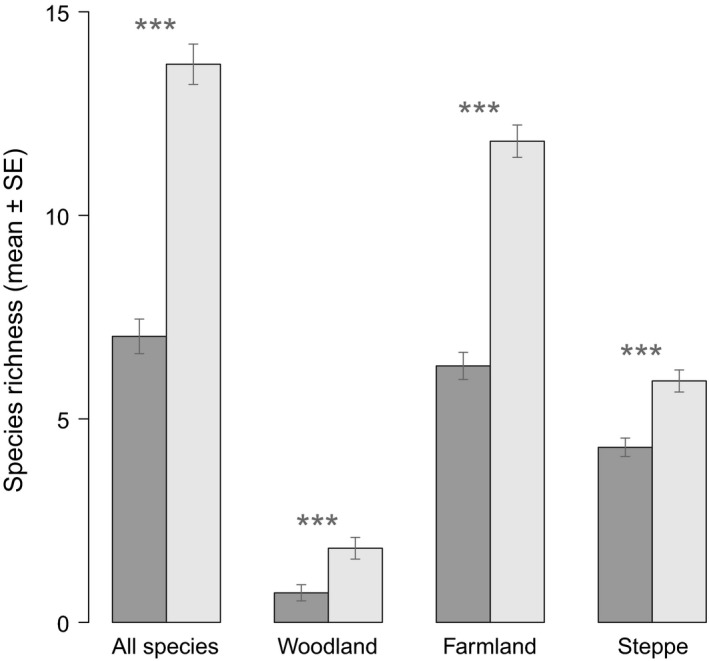
Mean species richness (±*SE*) of bird assemblages (all species, woodland, farmland, and steppe) estimated in 250 m buffers around 73 transects, in 1995–1997 (dark gray bars) and in 2010–2012 (light gray bars). Significant differences (*p *<* *.001; paired *t*‐tests) between time periods are marked with***

On average, the highest species richness per transect was found for farmland and steppe birds, while there were relatively few woodland species (Figure 3). The mean species richness of overall, woodland, and farmland bird assemblages doubled between 1995–1997 and 2010–2012, while the temporal increase in steppe bird species richness was small, albeit statistically significant (Figure 3). Farmland and steppe birds occurred in nearly every transect in both periods, whereas the prevalence of woodland birds increased from 30% to 60%.

Landscape composition was strongly dominated by the production component, though with marked temporal changes in the relative importance of land cover categories (Table 1). There were strong decreases in cover by annual dry crops, arable land with scattered trees, and annual irrigated crops, and increases in permanent pastures and permanent crops. The natural component occupied a much smaller proportion of the landscape, and it was mainly represented by woodlands and open woodlands (Table 1). Only the cover by water bodies changed significantly (increased) over time. Landscape heterogeneity varied little over time, although there was a reduction in the compositional heterogeneity of the production component, with significant declines in land cover diversity and evenness (Table 1).

**Figure 4:**
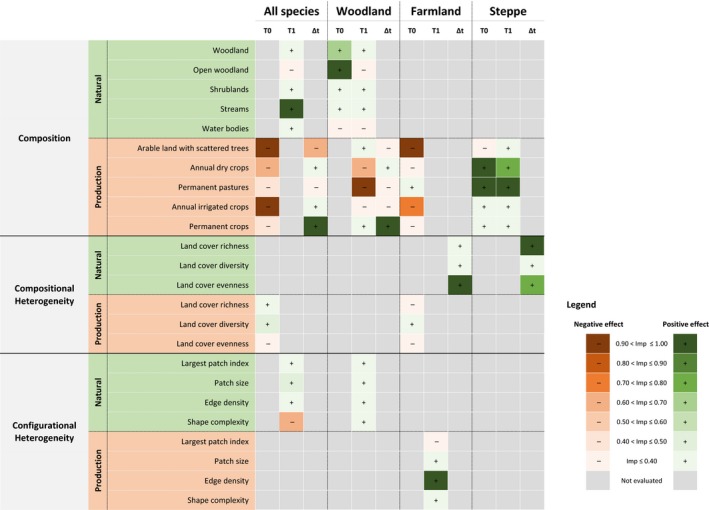
Graphical representation of the relative importance of landscape variables to explain spatial (T0 = 1995–1997, T1 = 2010–2012) and temporal (∆t) variation in bird species richness in farmland landscapes of southern Portugal. The importance of landscape variables was estimated from average models built separately for each of four bird assemblages (all species, woodland, farmland, and steppe). The variables used in modeling reflect composition, compositional heterogeneity, and configurational heterogeneity, of the natural and production components of the landscapes

There was strong support for landscape effects on spatial and temporal variation in species richness, with one to three sets of landscape variables showing summed Akaike weights >0.50 in the models for different time periods and species groups (Table 2). Average models further confirmed strong effects of individual landscape variables (Figure 4), although their explanatory power was much higher for spatial (*R* ^2^: 0.15 − 0.78) than for temporal (*R* ^2^: 0.06 − 0.25) variations (Tables S4–S6). Spline correlograms pointed out strong spatial autocorrelation in the raw data, but that this was successfully removed by the landscape models, as there was no significant autocorrelation in the residuals (Figures S2–S5).

**Table 2:** Relative importance of sets of variables describing composition, compositional heterogeneity, and configurational heterogeneity of either the natural or production components of the landscape, to explain spatial (T0: 1995–1997 and T1: 2010–2012) and temporal (Δt) variation in bird species richness in farmland landscapes of southern Portugal

Variable set	All species			Woodland			Farmland			Steppe		
	T0	T1	Δt	T0	T1	Δt	T0	T1	Δt	T0	T1	Δt
**Composition**												
Natural component	0.05	**0.69**	0.02	**0.70**	**0.96**	0.00	0.02	0.12	0.03	0.01	0.02	0.01
Production component	**1.00**	0.28	**0.96**	0.02	**1.00**	**0.99**	**1.00**	0.03	0.22	**0.99**	**0.96**	0.07
**Compositional heterogeneity**												
Natural component	0.10	0.32	0.26	0.22	0.04	0.03	0.19	0.14	**0.65**	0.14	0.03	**0.94**
Production component	**0.76**	0.26	0.35	0.14	0.07	0.08	**0.56**	0.06	0.27	0.04	0.04	0.18
**Configurational heterogeneity**												
Natural component	0.06	**0.53**	0.02	0.35	**0.99**	0.05	0.13	0.25	0.04	0.05	0.06	0.04
Production component	0.12	0.12	0.01	0.25	0.00	0.03	0.07	**0.63**	0.03	0.02	0.02	0.05

The importance of each set of variables was estimated as the sum of Akaike weights (*w*_i_+) of candidate models where that set occurs, considering a pool of 63 candidate models involving all combinations of sets of variables. Sets with w_i_+ > 0.5 were carried over to subsequent analysis and are given in bold.

### 3.2 Effects of landscape composition

In line with expectations, the composition of the natural component contributed to explain spatial variation in total species richness in 2010–2012 (w_*i*_+ = 0.69) and that of woodland birds in both periods (w_*i*_+ = 0.70 and 0.96), but did not influence farmland and steppe birds (Table 2). Total species richness in 2010–2012 increased with increasing cover by streams (Figure 4, Table S5). The richness of woodland birds increased along with cover by woodland and open woodland in 1995–1997, but no individual variable was particularly important in 2010–2012 (Figure 4, Tables S4 and S5). Temporal variation in species richness was little affected by the composition of the natural component (Figure 4, Table S6).

Also in line with our expectations, the composition of the production component was an important predictor of spatial and temporal variation in species richness (Table 2). The effects on spatial variation were particularly marked for total species richness (w_*i*_+ = 1.00) and that of farmland (1.00) and steppe birds (0.99) in 1995–1997 and for species richness of woodland (0.99) and steppe birds (0.96) in 2010–2012 (Table 2). All production cover categories were negatively related to total species richness in 1995–1997, albeit with much stronger effects of arable land with scattered trees and annual irrigated crops (Figure 4, Table S4). Permanent pastures and annual dry crops had negative effects on woodland birds in 2010–2012 and positive effects on steppe birds in both periods (Figure 4, Tables S4 and S5). Arable land with scattered trees and annual irrigated crops were negatively related to the richness of farmland birds in 1995–1997 (Figure 4, Table S4). The composition of the production component had particularly marked effects on the temporal variation of total (w_*i*_+ = 0.96) and woodland (1.00) bird species richness (Table 2). For both groups, richness was positively related with cover by permanent crops, and the total species richness also increased with declining cover by arable land with scattered trees (Figure 4, Table S6).

### 3.3 Effects of compositional and configurational landscape heterogeneity

According to our expectations, we found some effects of both compositional and configurational heterogeneity on species richness, although these effects were generally weaker than those of landscape composition (Table 2). We also found some evidence that heterogeneity of the natural component had stronger effects on woodland than on farmland and steppe bird species, and the opposite for the heterogeneity of the production component, although the effects were generally weak and partly inconsistent (Table 2).

Regarding the natural component, the compositional heterogeneity did not influence spatial variation in species richness, but configurational heterogeneity contributed to woodland (w_*i*_+ = 0.99) and, to a much lesser extent, total (w_*i*_+ = 0.53) bird species richness in 2010–2012 (Table 2). Total species richness increased along with patch size and declined with shape complexity (Figure 4, Table S5), while there was a weak tendency for woodland bird richness to increase with patch size (Figure 2, Table S9). Compositional heterogeneity contributed to temporal variations in farmland (w_*i*_+ = 0.73) and steppe (w_*i*_+ = 0.95) bird species richness (Table 2). The richness of steppe birds increased with the richness and evenness of natural cover categories, whereas the later was also positively related to farmland bird richness (Figure 4, Table S6).

Heterogeneity of the production component had weak to no effects on spatial variation in species richness and no effects on temporal variations (Figure 4, Table S4–S6). The compositional heterogeneity contributed moderately to variation in total species richness in 1995–1997 (w_*i*_+ = 0.68; Table 2), when it increases along with crop diversity (Figure 4, Table S4). The configurational heterogeneity contributed moderately to farmland bird species richness in 2010–2012 (w_*i*_+ = 0.64; Table 2), when there was a positive effect of edge density (Figure 4, Table S5).

## 4 Discussion

Our study examined the relative role of landscape composition and heterogeneity on spatial and temporal variations in avian diversity in Mediterranean farmland, showing that the composition of the natural and the production components had far stronger effects than those of their compositional or configurational heterogeneity (sensu Fahrig et al., 2011). Specifically, our study supported the expectation that the natural component should have a strong effect on species richness, in particular that of woodland and shrubland birds, while the effects of the production component should also be strong, particularly on farmland and steppe bird species. In contrast, the effects of heterogeneity were relatively weak and inconsistent, with few clear relationships between species richness and variables describing the diversity of land cover types (i.e., compositional heterogeneity) or the spatial arrangement of such cover types (i.e., configurational heterogeneity). These results might be seen as surprising, considering the prominent role given to heterogeneity as a key driver of farmland biodiversity (Benton et al., 2003; Fahrig et al., 2011), but they are consistent with a vast literature pointing out the strong effects of crop type and management (Berg et al., 2015; Butler et al., 2010; Chamberlain et al., 2001; Hiron et al., 2015; Josefsson et al. 2016; Rey, 2011; Stoate et al., 2009; Wilson et al., 2005). Overall, therefore, our results suggest that both composition and heterogeneity should be duly considered when managing farmland landscapes for conservation, with a particular emphasis on the identity and amount of different crop types because these may have far reaching consequences on species richness.

### 4.1 The natural component of the landscape benefited avian diversity

The expectation that avian diversity is strongly shaped by the composition of the natural component of the landscape was mainly supported by the positive relation between streams and overall species richness and between woodlands and the richness of woodland/shrubland species. Streams covered only a very small proportion of the landscape, but they were important possibly because they were often associated with arboreal and shrubby riparian galleries, which tend to be occupied by a number of woodland, shrubland, and specialized riparian species that are absent in surrounding open farmland (Pereira, Godinho, Gomes, & Rabaça, 2014). Transects close to streams thus sampled those species, together with more typical farmland species, thereby justifying their positive influence on overall diversity. It is worth noting, however, that streams were only influential after the CAP reform of 2003, when there was a marked increase in the pool of woodland/shrubland species in the study area (Santana et al., 2014; this study).

In contrast to streams, woodlands favored the richness of woodland/shrubland species but were poor predictors of overall diversity, although they are known to be species‐rich habitats (Santana, Porto, Gordinho, Reino, & Beja, 2012), and diversity tends to increase with the size of woodland patches (Santos, Tellería, & Carbonell, 2002). However, woodlands tend to be unsuitable for a range of farmland species, particularly steppe birds due to habitat loss and edge effects (Batáry, Fischer, Báldi, Crist, & Tscharntke, 2011; Concepción & Díaz, 2011; Fischer et al., 2011; Moreira et al., 2012; Morgado et al., 2010; Reino et al., 2009) and so there was probably a trade‐off between increases in woodland species and declines in some farmland species.

### 4.2 Composition of the production component was key to avian diversity

Also in line with expectations, the composition of the production component showed strong effects on species richness. Effects were generally stronger on farmland and steppe birds, probably because they often live within the production area, and so they should be particularly affected by the identity and amount of different crop types represented in farmland landscapes (Berg et al., 2015; Butler et al., 2010; Chamberlain et al., 2001; Hiron 2015; Josefsson et al. 2016; Rey, 2011; Stoate et al., 2009; Wilson et al., 2005). This is illustrated by the strong negative effects of cover by annual irrigated crops on the species richness of farmland birds observed in 1995–1997 that was probably a consequence of these crops providing poor breeding and foraging habitats for a range of species (Brotons, Mañosa, & Estrada, 2004; Stoate et al., 2009). The negative effects of arable land with scattered trees probably reflect the same mechanism, as this land cover type was often associated with the production of annual irrigated crops. The species richness of steppe birds was positively affected by the amount of annual dry crops and permanent pastures in both study periods, probably because most of these species are associated with these habitat types (Delgado & Moreira, 2000; Moreira, 1999; Reino et al., 2009, 2010; Stoate et al., 2000).

The composition of the production component also affected the overall diversity, but this was probably mediated to a considerable extent by the effects on farmland birds, which are the dominant group in the region. For instance, the negative relationship observed between total species richness and cover by arable land with scattered trees and by annual irrigated crops was probably a consequence of the strongly negative effect of these habitats on farmland birds. However, the production component also affected nonfarmland birds, which was clearly underlined by the positive effects of permanent crops on the spatial (in 2010–2012) and temporal increase in woodland bird species richness. Permanent crops in our area were mainly olive orchards, which have structural similarities with woodlands, and may thus attract species that otherwise would be rare or absent in open arable farmland (Rey, 2011). As a consequence, cover by permanent crops showed strongly positive effects on total species richness, although these habitats are known to be avoided by a range of steppe birds associated with open farmland habitats (Stoate et al., 2009).

Despite the strong effects of the production component, the influential crops varied between study periods, which was probably a consequence of the major changes in agricultural land uses associated with the CAP reform of 2003 (Ribeiro et al., 2014; Santana et al., 2014). This is illustrated by the permanent crops, which were only influential after the CAP reform, when they became a dominant land cover type (Ribeiro et al., 2014). In contrast, the influence of annual arable crops was only evident in 1995–1997, before their representation in the landscape declined markedly possibly due to the changes associated with the CAP reform (Ribeiro et al., 2014). Overall, these results suggest that the influence of different crop types may change over time and that this may be related to their prevalence across the landscape.

### 4.3 Avian diversity was weakly related to landscape heterogeneity

As expected (Fahrig et al., 2011), landscape compositional and configurational heterogeneity had some effects on avian diversity, but these were relatively weak and inconsistent. Nevertheless, there was a tendency in 1995–1997 for total bird diversity increasing with the diversity of crop types, which is consistent with the idea that the presence of different habitats benefits biodiversity by providing conditions for a wide range of species with contrasting ecological requirements (Benton et al., 2003; Fahrig et al., 2011; Fuller, Hinsley, & Swetnam, 2004). This is also supported to some extent by the positive effects of cover richness and evenness of the natural component on the temporal variation of farmland and steppe bird species richness, although these results are difficult to interpret because these species are mainly associated with crop habitats (Moreira et al., 2012; Morgado et al., 2010; Reino et al., 2009, 2010), and the explanatory power of models including these variables was small (*R* ^2^: 0.05 − 0.12). In contrast to these results, the total species richness in 2010–2012 seemed to be negatively affected by the configurational heterogeneity of the natural component, as there was a positive relation with patch size and a negative relation with patch complexity. This suggests that diversity was benefited by large patches of natural habitat, possibly due to species–area effects (Fischer & Lindenmayer, 2002), rather than heterogeneity per se.

The contrast between our results and the importance normally given to heterogeneity on farmland may be a consequence of some particularities of our study, although it may also reflect some general patterns applying to farmland landscapes. First, we used relatively coarse land cover categories, which were designed to have management relevance and to encompass a large pool of bird species with different habitat requirements, although a more detailed habitat categorization might be needed to perceive finer responses to landscape heterogeneity (Fahrig et al., 2011). This is supported to some extent by previous studies in our area showing that species richness often peaked close to the edges (Reino et al., 2009) and that different habitat types are needed to provide conditions for diverse steppe bird assemblages (Reino et al., 2010). Therefore, the influence of heterogeneity may have been underestimated somewhat, although this is unlikely to have affected the strong effects observed for landscape composition. Second, our study may have represented a relatively limited range of variation in landscape heterogeneity, because we sampled areas that were largely dominated by homogeneous open arable land, particularly before the CAP reform of 2003, with virtually no hedgerows and only relatively small woodland and shrubland patches. This may have emphasized the importance of landscape composition, as the production component showed marked spatial and temporal variations (Ribeiro et al., 2014). Finally, the results may have been influenced by the particular species pool occurring in our study area, which included many specialized species associated with large and relatively homogeneous expanses of open farmland habitat (Moreira et al., 2012; Morgado et al., 2010; Reino et al., 2009, 2010) that are typical of similar landscapes across the Iberian Peninsula (e.g., Concepción & Díaz, 2011). Therefore, heterogeneity may have had a positive influence on some species but negative on others, thereby reducing its overall effects. Whatever the reasons, however, our results point out that the importance of heterogeneity may vary across farmland landscapes, probably depending on local ecological characteristics and agricultural land uses.

## 5 Conclusions

There are increasing efforts to promote the conservation of biodiversity on farmland while minimizing impacts on economic output, and enhancing landscape heterogeneity has been recommended as a key solution to achieve this goal (Fahrig et al., 2011). Our results suggest that this option may not be adequate in every case, because farmland diversity in at least some landscapes may be far more affected by the identity of crops produced, rather than by their diversity or spatial configuration. Although this view results from a specific case study focusing on particular ecological and agricultural conditions, it is in line with a wealth of research showing strong links between biodiversity and the type and management of crops (Berg et al., 2015; Butler et al., 2010; Chamberlain et al., 2001; Hiron 2015; Josefsson et al. 2016; Rey, 2011; Stoate et al., 2009; Wilson et al., 2005). Therefore, we suggest that the composition of the production component of the landscape needs to be carefully considered when managing farmland for biodiversity, particularly in ours and other open Mediterranean farmland landscapes where there is a range of species tightly associated with crops and pastures for breeding and foraging (Concepción & Díaz, 2011; Moreira et al., 2012; Reino et al., 2009, 2010). In our region, this implies maintaining large areas occupied by rain‐fed cereals, fallows, and extensive pastureland, which requires agricultural policies and agri‐environment subsidy schemes adjusted to local biophysical conditions and market demands (Ribeiro, Santos, Santana, Reino, Beja, et al., 2016; Ribeiro, Santos, Santana, Reino, Leitão, et al., 2016; Ribeiro et al., 2014; Santana et al., 2014). Overall, we suggest that future studies should explore these ideas in more detail, evaluating under what circumstances major benefits can be achieved by changing landscape heterogeneity (sensu Fahrig et al., 2011), and where such benefits require focusing primarily on what crops are grown and how they are managed.

## Conflict of Interest

None declared.
