# Supplementary material for: Combined effects of landscape composition and heterogeneity on farmland avian diversity
Source: Ecol Evol. 2017 Jan 25;7(4):1212–23. doi: 10.1002/ece3.2693 (PMC5306015; doi:10.1002/ece3.2693)
Supplement: Supplementary file 1 [file ECE3-7-1212-s001.pdf]

Supplementary material for:

Combined effects of landscape composition and heterogeneity on farmland avian diversity

Joana Santana, Luís Reino, Chris Stoate, Francisco Moreira, Paulo Flores Ribeiro, José Lima Santos, John T. Rotenberry, Pedro Beja

Table S1. Percentage of occurrence of bird species recorded in 73 transects sampled annually during the breeding season in southern Portugal, in 1995-1997 and 2010-2012. Species are classified according to their habitat affinities (F – farmland; S – steppe; W – woodland; O - other), conservation status (SPEC #), and phenology (R – resident, M – migratory).

Species[1]	Habitat affinities[2]	Conservation status[3]	Phenology	1995-1997	2010-2012
**Galliformes**					
Alectoris rufa	F	SPEC 2	R	16.44	47.95
Coturnix coturnix	F, S	SPEC 3	M	54.79	52.05
**Ciconiiformes**					
Bubulcus ibis	F		R	16.44	10.96
Ciconia nigra	W	SPEC 2	M	1.37	0
**Accipitriformes**					
Ciconia ciconia	F	SPEC 2	R,M	15.07	32.88
Elanus caeruleus	F	SPEC 3	R	1.37	8.22
Milvus migrans	F	SPEC 3	M	4.11	8.22
Milvus milvus	F	SPEC 2	M	0	2.74
Gyps fulvus	F		R	0	1.37
Circaetus gallicus	W	SPEC 3	M	0	4.11
Circus aeruginosus	O		M	0	2.74
Circus pygargus	F, S		M	23.29	31.51
Buteo buteo	F		R	1.37	12.33
Aquila adalberti	W	SPEC 1	R	0	4.11
Aquila pennata	W	SPEC 3	M	0	1.37
Aquila fasciata	F	SPEC 3	R	0	2.74
Falco naumanni	F, S	SPEC 1	M	1.37	27.4
Falco tinnunculus	F	SPEC 3	R	1.37	10.96
**Gruiformes**					
Tetrax tetrax	F, S	SPEC 1	R	54.79	56.16
Otis tarda	F, S	SPEC 1	R	19.18	23.29
**Charadriiformes**					
Burhinus oedicnemus	F, S	SPEC 3	R	8.22	21.92
Glareola pratincola	F, S	SPEC 3	M	0	1.37
**Pteroclidiformes**					
Pterocles orientalis	F, S	SPEC 2	R	0	12.33
**Columbiformes**					
Columba livia	F		R	0	2.74
Columba palumbus	W		R	0	12.33
Streptopelia decaocto	F		R	0	21.92
Streptopelia turtur	F	SPEC 3	M	0	1.37
Clamator glandarius	F		M	1.37	8.22
Cuculus canorus	F		M	5.48	6.85
**Strigiformes**					
Athene noctua	F	SPEC 3	R	5.48	6.85

Species[1]	Habitat affinities[2]	Conservation status[3]	Phenology	1995-1997	2010-2012
**Coraciiformes**					
Merops apiaster	F	SPEC 3	M	12.33	52.05
Coracias garrulus	F, S	SPEC 2	M	0	4.11
**Piciformes**					
Dendrocopos major	W		R	0	1.37
**Passeriformes**					
Melanocorypha calandra	F, S	SPEC 3	R	30.14	36.99
Calandrella brachydactyla	F, S	SPEC 3	M	38.36	34.25
Galerida spp. *	F, S	SPEC 3	R	21.92	75.34
Lullula arborea	W	SPEC 2	R	10.96	9.59
Hirundo rustica	F	SPEC 3	M	19.18	43.84
Cecropis daurica	F		M	0	4.11
Delichon urbicum	F	SPEC 3	M	0	5.48
Anthus campestris	F, S	SPEC 3	M	4.11	20.55
Motacilla flava	F		M	0	4.11
Motacilla alba	F		R	1.37	1.37
Cercotrichas galactotes	W	SPEC 3	M	0	1.37
Luscinia megarhynchos	W		M	4.11	15.07
Saxicola rubicola	F		R	19.18	35.62
Oenanthe hispanica	F, S	SPEC 2	M	13.7	21.92
Turdus viscivorus	W		R	0	1.37
Turdus merula	W		R	8.22	34.25
Upupa epops	F	SPEC 3	M	24.66	26.03
Cettia cetti	W		R	4.11	4.11
Cisticola juncidis	F, S		R	65.75	80.82
Acrocephalus scirpaceus	O		M	0	1.37
Acrocephalus arundinaceus	O		M	0	1.37
Hippolais polyglotta	W		M	0	2.74
Sylvia atricapilla	W		R	0	1.37
Sylvia hortensis	F	SPEC 3	M	0	1.37
Sylvia undata	W	SPEC 2	R	0	2.74
Sylvia cantillans	W		M	0	2.74
Sylvia melanocephala	W		R	12.33	15.07
Phylloscopus ibericus	W		M	0	1.37
Phyloscopus collybita	W		M	0	1.37
Aegithalos caudatus	W		R	1.37	0
Cyanistes caeruleus	W		R	8.22	13.7
Parus major	W		R	12.33	10.96
Certhia brachydactyla	W		R	2.74	8.22
Oriolus oriolus	W		M	0	1.37
Lanius meridionalis	F		R	12.33	20.55
Lanius senator	F	SPEC 2	M	15.07	8.22
Garrulus glandarius	W		R	0	4.11
Cyanopica cyanus	W		R	0	21.92
Pica pica	F		R	0	10.96

Species[1]	Habitat affinities[2]	Conservation status[3]	Phenology	1995-1997	2010-2012
Corvus monedula	F		R	0	2.74
Corvus corone	F		R	0	26.03
Corvus corax	W		R	4.11	4.11
Sturnus unicolor	F		R	9.59	28.77
Passer spp.**	F		R	8.22	34.25
Fringila coelebs	W		R	2.74	1.37
Serinus serinus	F		R	0	4.11
Chloris chloris	F		R	4.11	20.55
Carduelis carduelis	F		R	5.48	49.32
Carduelis cannabina	F	SPEC 2	R	0	23.29
Estrilda astrild	O		R	0	1.37
Emberiza calandra	F, S	SPEC 2	R	94.52	93.15

[1] Species are listed in taxonomic order following Equipa Atlas (2008).

[2] Bird habitat categorizations based on Ehrlich *et al.* (1994), Suárez *et al.* (1997), Equipa Atlas (2008), Reino *et al.* (2009) and EBCC (2012).

[3] Species of European Conservation Concern: SPEC 1 - Species of global conservation concern; SPEC 2 - species concentrated in Europe and with an unfavorable conservation status; SPEC 3 - species not concentrated in Europe but with an unfavorable conservation status (BirdLife International 2004).

* *Galerida* spp.: includes *Galerida theklae*, *G. cristata* and *Galerida* sp. observations.

** *Passer* spp.: includes *Passer domesticus*, *P. hispaniolensis* and *Passer* sp. observations. We have not considered *Passer* spp. as a SPEC species because most of the identified records were from *P. hispaniolensis*.

Table S2. Description of variables used to quantify landscape compositional and configurational heterogeneity in 250-m buffers around 73 transects used to estimate bird species richness in 1995-1997 and 2010-2012, in southern Portugal.

Landscape variable (unit, abbreviation)	Description
**Compositional heterogeneity**	
Land cover richness (no., CR)	Total number of different natural/production land cover types.
Land cover diversity (SHDI)[a]	Shannon's diversity index computed on the proportion of different natural/production land cover types.
Land cover evenness (SHEI)[b]	Shannon's evenness index computed on the proportion of different natural/production land cover types.
**Configurational heterogeneity**	
Largest patch index (%, LPI)	Percentage of area of the largest natural/production land cover type patch.
Patch size (ha, AREA)	Mean area of natural/production land cover type patches.
Edge density (m^2/ha, ED)	Density of edges between natural and production land cover type patches.
Shape complexity (SHAPE)	Mean perimeter-to-area ratio of natural/production land cover type patches.

[a]SHDI = 0 when the landscape contains only 1 or 0 cover types;

[b]SHEI = 0 when the landscape contains only 1 or 0 cover types. SHEI = 1 when distribution of area among patch types is perfectly even (i.e., proportional abundances are the same).

Table S3. Formulation of candidate models ($g_{1\text{-}63}$) based on all possible combinations of the six sets of landscape variables listed in Table 1.

No. variable sets	No. models	Model formulation
One set	6	g_1 = Set 1
		g_2 = Set 2
		g_3 = Set 3
		g_4 = Set 4
		g_5 = Set 5
		g_6 = Set 6
Two sets	15	g_7 = Set 1 + Set 2
		g_8 = Set 1 + Set 3
		g_9 = Set 1 + Set 4
		g_{10} = Set 1 + Set 5
		g_{11} = Set 1 + Set 6
		g_{12} = Set 2 + Set 3
		g_{13} = Set 2 + Set 4
		g_{14} = Set 2 + Set 5
		g_{15} = Set 2 + Set 6
		g_{16} = Set 3 + Set 4
		g_{17} = Set 3 + Set 5
		g_{18} = Set 3 + Set 6
		g_{19} = Set 4 + Set 5
		g_{20} = Set 4 + Set 6
		g_{21} = Set 5 + Set 6
Three sets	20	g_{22} = Set 1 + Set 2 + Set 3
		g_{23} = Set 1 + Set 2 + Set 4
		g_{24} = Set 1 + Set 2 + Set 5
		g_{25} = Set 1 + Set 2 + Set 6
		g_{26} = Set 1 + Set 3 + Set 4
		g_{27} = Set 1 + Set 3 + Set 5
		g_{28} = Set 1 + Set 3 + Set 6
		g_{29} = Set 1 + Set 4 + Set 5
		g_{30} = Set 1 + Set 4 + Set 6

No. variable sets	No. models	Model formulation
		g_{31} = Set 1 + Set 5 + Set 6
		g_{32} = Set 2 + Set 3 + Set 4
		g_{33} = Set 2 + Set 3 + Set 5
		g_{34} = Set 2 + Set 3 + Set 6
		g_{35} = Set 2 + Set 4 + Set 5
		g_{36} = Set 2 + Set 4 + Set 6
		g_{37} = Set 2 + Set 5 + Set 6
		g_{38} = Set 3 + Set 4 + Set 5
		g_{39} = Set 3 + Set 4 + Set 6
		g_{40} = Set 3 + Set 5 + Set 6
		g_{41} = Set 4 + Set 5 + Set 6
Four sets	15	g_{42} = Set 1 + Set 2 + Set 3 + Set 4
		g_{43} = Set 1 + Set 2 + Set 3 + Set 5
		g_{44} = Set 1 + Set 2 + Set 3 + Set 6
		g_{45} = Set 1 + Set 2 + Set 4 + Set 5
		g_{46} = Set 1 + Set 2 + Set 4 + Set 6
		g_{47} = Set 1 + Set 2 + Set 5 + Set 6
		g_{48} = Set 1 + Set 3 + Set 4 + Set 5
		g_{49} = Set 1 + Set 3 + Set 4 + Set 6
		g_{50} = Set 1 + Set 3 + Set 5 + Set 6
		g_{51} = Set 1 + Set 4 + Set 5 + Set 6
		g_{52} = Set 2 + Set 3 + Set 4 + Set 5
		g_{53} = Set 2 + Set 3 + Set 4 + Set 6
		g_{54} = Set 2 + Set 3 + Set 5 + Set 6
		g_{55} = Set 2 + Set 4 + Set 5 + Set 6
		g_{56} = Set 3 + Set 4 + Set 5 + Set 6
Five sets	6	g_{57} = Set 1 + Set 2 + Set 3 + Set 4 + Set 5
		g_{58} = Set 1 + Set 2 + Set 3 + Set 4 + Set 6
		g_{59} = Set 1 + Set 2 + Set 3 + Set 5 + Set 6
		g_{60} = Set 1 + Set 2 + Set 4 + Set 5 + Set 6
		g_{61} = Set 1 + Set 3 + Set 4 + Set 5 + Set 6
		g_{62} = Set 2 + Set 3 + Set 4 + Set 5 + Set 6
Six sets	1	g_{63} = Set 1 + Set 2 + Set 3 + Set 4 + Set 5 + Set 6

Table S4. Summary of average models relating spatial variation in bird species richness in 1995-1997 to landscape variables. In each case we provide the model-averaged partial standardized coefficients (Coef) and their partial standardized standard error (SE). The relative importance of each variable in the model (Imp) was calculated as the ratio between the respective partial standardized coefficient and the largest standardized coefficient in the model (Cade 2015). Variables are ordered by their relative importance within each model. Variables with Imp > 0.4 are in bold, and the ones with negative effects are underlined. See main text for methodological details.

Variable set	Landscape variable	Coef	SE	Imp
**All species (R^2 = 0.58)**				
**Composition/Production**	**Arable land with scattered trees**	**-0.33**	**0.06**	**1.00**
**Composition/Production**	**Irrigated annual crops**	**-0.30**	**0.06**	**0.91**
**Composition/Production**	**Annual dry crops**	**-0.23**	**0.05**	**0.70**
**Composition/Production**	**Permanent pastures**	**-0.18**	**0.05**	**0.55**
**Compositional heterogeneity/Production**	**Cover diversity (Production)**	**0.14**	**0.06**	**0.43**
**Composition/Production**	**Permanent crops**	**-0.14**	**0.06**	**0.42**
Compositional heterogeneity/Production	Cover evenness (Production)	-0.10	0.06	0.31
Compositional heterogeneity/Production	Cover richness (Production)	0.00	0.03	0.00
**Woodland birds (R^2 = 0.78)**				
**Composition/Natural**	**Open woodland**	**0.71**	**0.11**	**1.00**
**Composition/Natural**	**Woodland**	**0.43**	**0.07**	**0.61**
Composition/Natural	Water bodies	-0.08	0.15	0.11
Composition/Natural	Streams	0.04	0.08	0.06
Composition/Natural	Shrubland	0.03	0.08	0.04
**Farmland birds (R^2 = 0.39)**				
**Composition/Production**	**Arable land with scattered trees**	**-0.24**	**0.07**	**1.00**
**Composition/Production**	**Annual irrigated crops**	**-0.17**	**0.07**	**0.72**
Compositional heterogeneity/Production	Cover diversity (Production)	0.09	0.07	0.37
Compositional heterogeneity/Production	Cover evenness (Production)	-0.06	0.07	0.23
Composition/Production	Permanent crops	-0.04	0.06	0.18
Composition/Production	Dry annual crops	-0.03	0.05	0.12
Composition/Production	Permanent pastures	0.01	0.03	0.02
Compositional heterogeneity/Production	Cover richness (Production)	-0.01	0.04	0.02
**Steppe birds (R^2 = 0.31)**				
**Composition/Production**	**Permanent pastures**	**0.19**	**0.07**	**1.00**
**Composition/Production**	**Annual dry crops**	**0.19**	**0.06**	**0.99**
Composition/Production	Arable land with scattered trees	-0.03	0.06	0.15
Composition/Production	Annual irrigated crops	0.01	0.04	0.06
Composition/Production	Permanent crops	0.01	0.04	0.06

Table S5. Summary of average models relating spatial variation in bird species richness in 2010-2012 to landscape variables. In each case we provide the model-averaged partial standardized coefficients (Coef) and their partial standardized standard error (SE). The relative importance of each variable in the model (Imp) was calculated as the ratio between the respective partial standardized coefficient and the largest standardized coefficient in the model (Cade 2015). Variables are ordered by their relative importance within each model. Variables with Imp > 0.4 are in bold, and the ones with negative effects are underlined. See main text for methodological details.

Variable set	Landscape variable	Coef	SE	Imp
**All species (R^2 = 0.38)**				
**Composition/Natural**	**Streams**	**0.08**	**0.04**	**1.00**
**Configurational heterogeneity/Natural**	**Shape complexity (Natural)**	**-0.05**	**0.05**	**0.61**
**Configurational heterogeneity/Natural**	**Patch size (Natural)**	**0.04**	**0.04**	**0.44**
Configurational heterogeneity/Natural	Large patch index (Natural)	0.03	0.04	0.33
Composition/Natural	Woodland	0.02	0.03	0.28
Composition/Natural	Open woodland	-0.02	0.04	0.27
Composition/Natural	Shrubland	0.02	0.03	0.22
Configurational heterogeneity/Natural	Edge density (Natural)	0.01	0.03	0.07
Composition/Natural	Water bodies	0.00	0.02	0.03
**Woodland (R^2 = 0.76)**				
**Composition/Production**	**Permanent pastures**	**-0.66**	**0.26**	**1.00**
**Composition/Production**	**Annual dry crops**	**-0.40**	**0.21**	**0.61**
Configurational heterogeneity/Natural	Patch size (Natural)	0.26	0.11	0.39
Composition/Natural	Woodland	0.17	0.11	0.26
Composition/Natural	Shrubland	0.16	0.11	0.24
Composition/Natural	Water bodies	-0.12	0.11	0.18
Composition/Natural	Open woodland	-0.10	0.10	0.15
Composition/Natural	Streams	0.07	0.10	0.11
Composition/production	Arable land with scattered trees	0.05	0.09	0.08
Composition/production	Permanent crops	0.05	0.15	0.07
Configurational heterogeneity/Natural	Large patch index (Natural)	0.02	0.08	0.03
Composition/Production	Annual irrigated crops	-0.01	0.09	0.02
Configurational heterogeneity/Natural	Shape complexity (Natural)	0.01	0.06	0.02
Configurational heterogeneity/Natural	Edge density (Natural)	0.01	0.06	0.01
**Farmland (R^2 = 0.15)**				
**Configurational heterogeneity/Production**	**Production edge density**	**0.09**	**0.05**	**1.00**
Configurational heterogeneity/Production	Mean production shape complexity	0.00	0.02	0.03
Configurational heterogeneity/Production	Largest production patch index	0.00	0.02	0.03
Configurational heterogeneity/Production	Mean patch area	0.00	0.02	0.03
**Steppe (R^2 = 0.29)**				
**Composition/Production**	**Permanent pastures**	**0.17**	**0.06**	**1.00**
**Composition/Production**	**Annual dry crops**	**0.14**	**0.06**	**0.79**
Composition/Production	Annual irrigated crops	0.03	0.05	0.16
Composition/Production	Arable land with scattered trees	0.02	0.04	0.14
Composition/Production	Permanent crops	0.00	0.04	0.01

Table S6. Summary of average models relating temporal variation in bird species richness to landscape variables. In each case we provide the model-averaged partial standardized coefficients (Coef) and their partial standardized standard error (SE). The relative importance of each variable in the model (Imp) was calculated as the ratio between the respective partial standardized coefficient and the largest standardized coefficient in the model (Cade 2015). Variables are ordered by their relative importance within each model. Variables with Imp > 0.4 are in bold, and the ones with negative effects are underlined. See main text for methodological details.

Variable set	Landscape variable	Coef	SE	Imp
**All species (R^2 = 0.17)**				
**Composition/production**	**Permanent crops**	**1.06**	**0.67**	**1.00**
**<u>Composition/production</u>**	**<u>Arable land with scattered trees</u>**	**<u>-0.65</u>**	**<u>0.63</u>**	**<u>0.61</u>**
Composition/production	Permanent pastures	-0.09	0.35	0.09
Composition/production	Annual dry crops	0.06	0.35	0.06
Composition/production	Annual irrigated crops	0.02	0.30	0.02
**Woodland (R^2 = 0.25)**				
**Composition/production**	**Permanent crops**	**0.62**	**0.26**	**1.00**
Composition/production	Permanent pastures	-0.13	0.21	0.21
Composition/production	Arable land with scattered trees	-0.08	0.15	0.13
Composition/production	Annual dry crops	0.05	0.20	0.08
Composition/production	Annual irrigated crops	-0.03	0.14	0.05
**Farmland (R^2 = 0.05)**				
**Compositional heterogeneity/Natural**	**Cover evenness (Natural)**	**0.56**	**0.56**	**1.00**
Compositional heterogeneity/Natural	Cover diversity (Natural)	0.09	0.41	0.17
Compositional heterogeneity/Natural	Cover richness (Natural)	0.00	0.25	0.00
**Steppe (R^2 = 0.12)**				
**Compositional heterogeneity/Natural**	**Cover richness (Natural)**	**0.37**	**0.35**	**1.00**
**Compositional heterogeneity/Natural**	**Cover evenness (Natural)**	**0.28**	**0.33**	**0.75**
Compositional heterogeneity/Natural	Cover diversity (Natural)	0.03	0.24	0.07

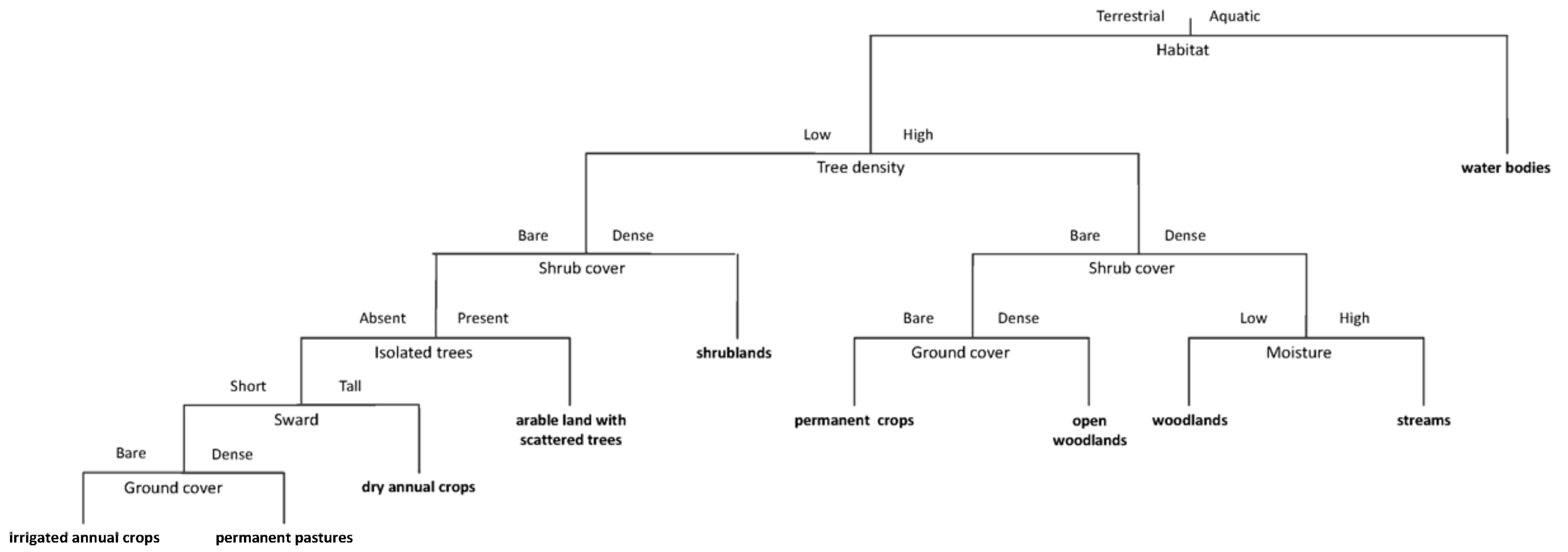

**Natural component**	
woodlands	= holm woods (*Quercus rotundifolia*) with >30% tree cover and shrub understory; old eucalyptus plantations with understory; tree lines
open woodlands	= holm woods with >30% tree cover and grassy understory, young plantations without understory
shrublands	= shrublands without trees or with scattered trees
streams	= streams with vegetation (herbs, shrubs or trees)
water bodies	= ponds and reservoirs
**Production component**	
arable land with scattered trees	= annual crops with scattered trees (<30% tree cover)
dry annual crops	= non-irrigated annual crops (dry cereals (e.g. wheat, barley) and legumes) in rotation with ploughed fields, stubbles and fallows usually grazed by sheep or cattle
permanent pastures	= grassy vegetation grazed by sheep or cattle
irrigated annual crops	= irrigated annual crops (corn and sunflower)
permanent crops	= permanent irrigated and non-irrigated crops (olive groves and vineyards)

Figure S1. Classification tree of land cover categories used to model the relations between bird species richness and landscape characteristics in southern Portugal. Categories were defined considering the main nesting and foraging habitats of bird species in the study area (Moreira 1999; Delgado & Moreira 2000; Stoate *et al.* 2000; Reino *et al.* 2009, 2010), and assuming that habitat preferences are often influenced strongly by structural characteristics (e.g. tree density, shrub cover, sward density and height, and amount of bare ground – ground cover). Characteristics of the herbaceous sward were considered during the sampling months (April-May), though they are known to vary strongly during the annual cycle (e.g., dry annual crops are sown in autumn and thus the sward is tall and dense during the breeding season, whereas irrigated annual crops are generally sown in spring, and so during the breeding season the sward tends to be short, sparse, and with a high proportion of bare ground).

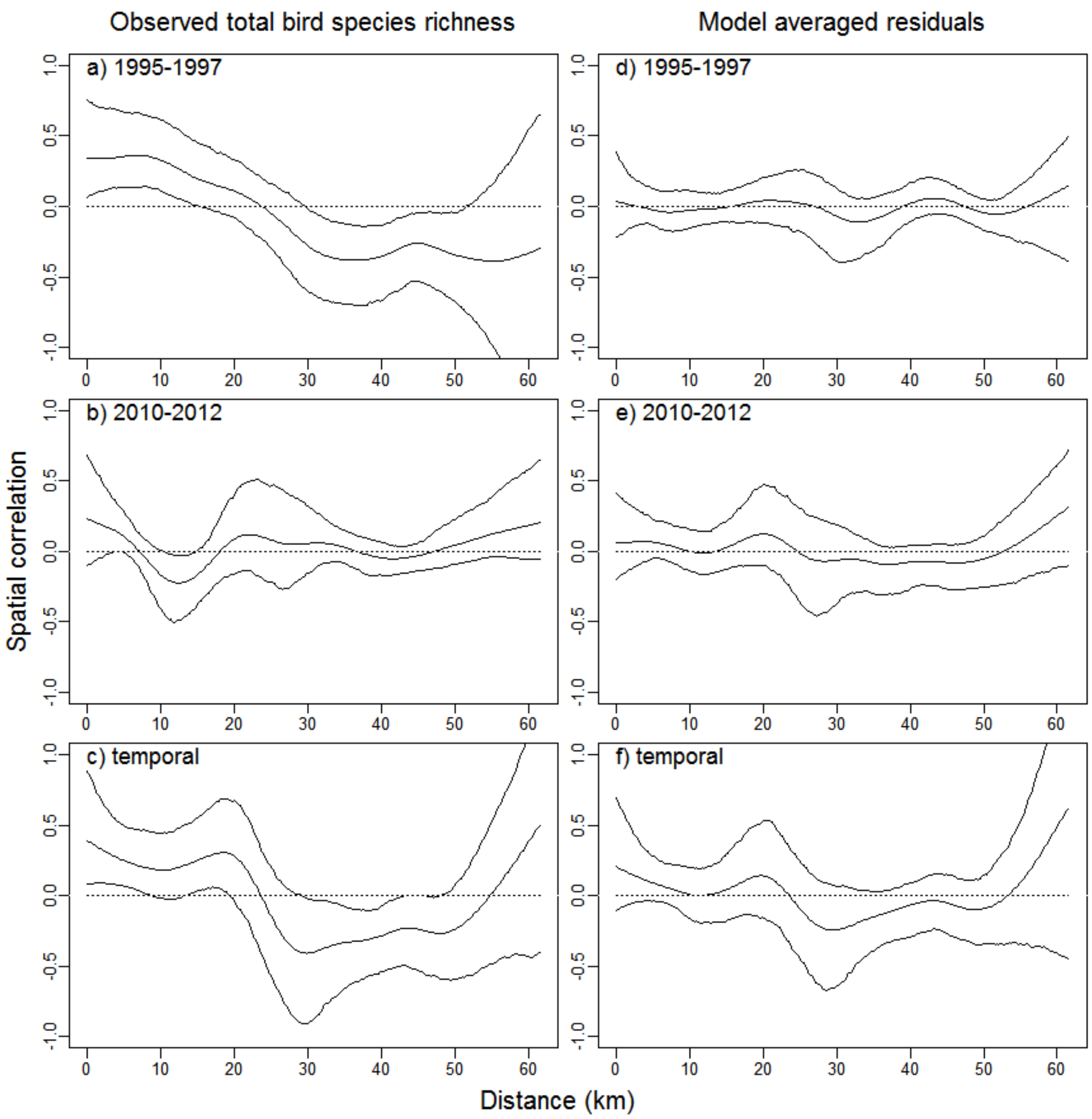

Figure S2. Spline correlograms describing spatial autocorrelation for total bird species richness and for the residuals of models relating species richness to landscape variables (Tables S4 – S6). Separate correlograms are presented for 1995-97 (a, d), 2010-12 (b, e), and temporal variation (c, f). Lines represent the estimate (in the middle) and the 95% confidence envelopes (external lines) using 1000 bootstrap resamples (Bjørnstad & Falck 2001).

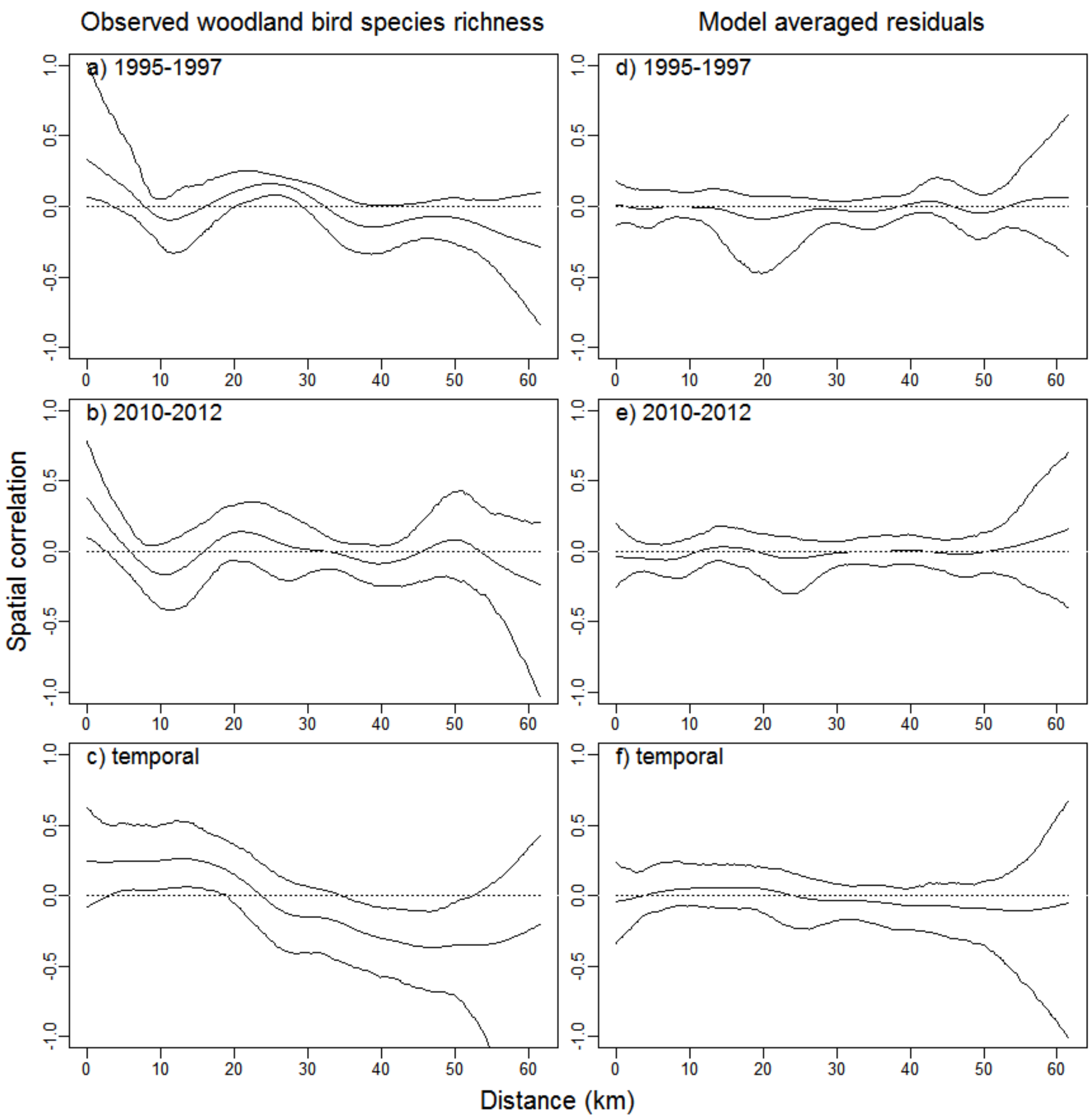

Figure S3. Spline correlograms describing spatial autocorrelation for woodland bird species richness and for the residuals of models relating species richness to landscape variables (Tables S4 – S6). Separate correlograms are presented for 1995-97 (a, d), 2010-12 (b, e), and temporal variation (c, f). Lines represent the estimate (in the middle) and the 95% confidence envelopes (external lines) using 1000 bootstrap resamples (Bjørnstad & Falck 2001).

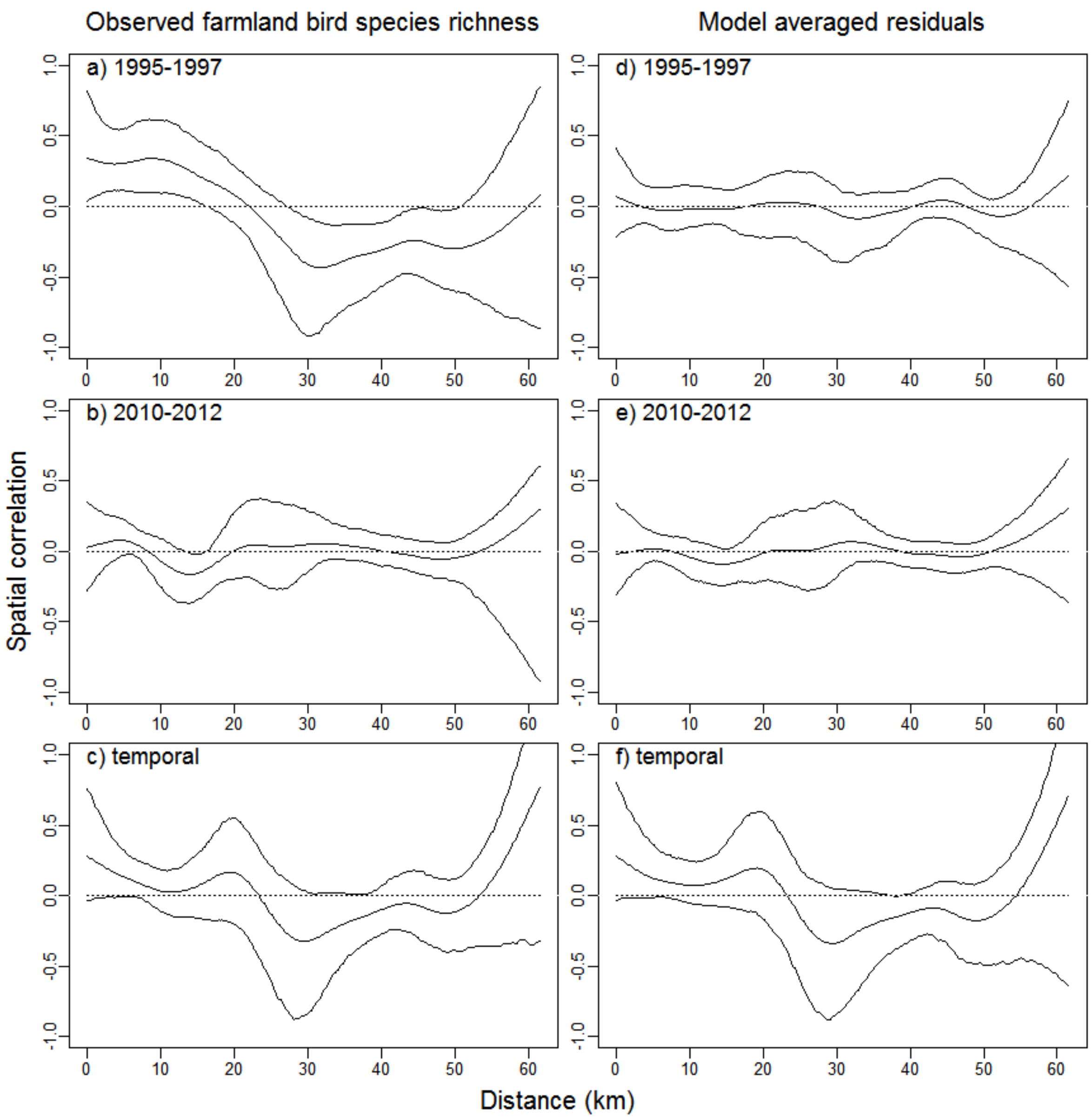

Figure S4. Spline correlograms describing spatial autocorrelation for farmland bird species richness and for the residuals of models relating species richness to landscape variables (Tables S4 – S6). Separate correlograms are presented for 1995-97 (a, d), 2010-12 (b, e), and temporal variation (c, f). Lines represent the estimate (in the middle) and the 95% confidence envelopes (external lines) using 1000 bootstrap resamples (Bjørnstad & Falck 2001).

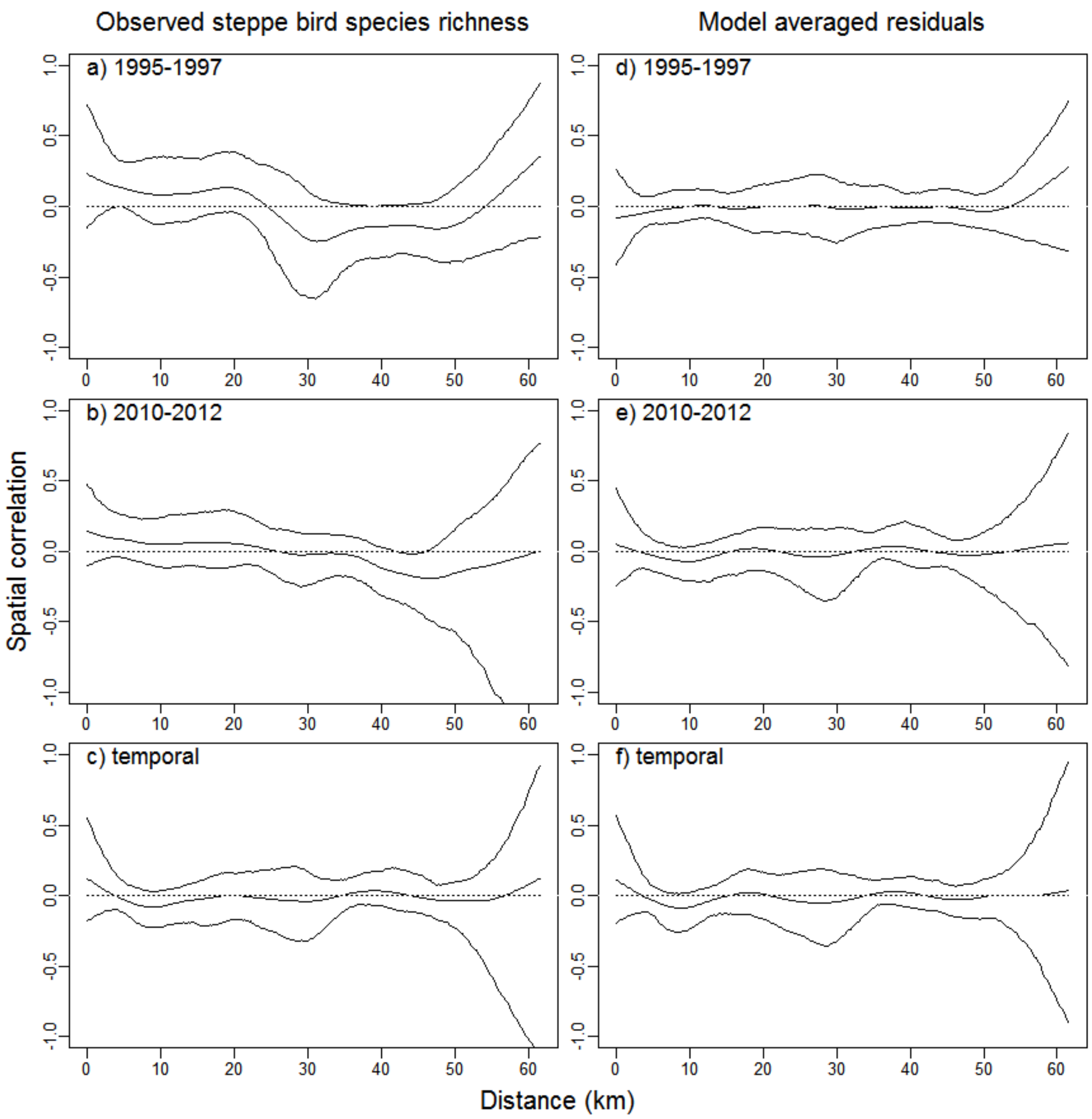

Figure S5. Spline correlograms describing spatial autocorrelation for steppe bird species richness and for the residuals of models relating species richness to landscape variables (Tables S4 – S6). Separate correlograms are presented for 1995-97 (a, d), 2010-12 (b, e), and temporal variation (c, f). Lines represent the estimate (in the middle) and the 95% confidence envelopes (external lines) using 1000 bootstrap resamples (Bjørnstad & Falck 2001).

Supporting references

BirdLife International (2004). *Birds in the European Union: a status assessment*. Wageningen, The Netherlands: BirdLife International.

Bjørnstad, O. N., & Falck, W. (2001). Nonparametric spatial covariance functions: estimation and testing. *Environmental and Ecological Statistics*, *8*, 53-70. doi:10.1023/A:1009601932481

Cade, B. S. (2015). Model averaging and muddled multimodel inferences. *Ecology, 96*, 2370-2382. doi:10.1890/14-1639.1

Delgado, A., & Moreira, F. (2000). Bird assemblages of an Iberian cereal steppe. *Agriculture, Ecosystems* & *Environment*, *78*, 65-76. doi:10.1016/S0167-8809(99)00114-0

EBCC (2012). http://www.ebcc.info/index.php?ID=294

Ehrlich, P.R., Dobkin, D.S., Wheye, D., & Pimm, S.L. (1994). *The Birdwatcher's Handbook: a guide to the Natural History of the Birds of Britain and Europe*. Oxford University Press, Oxford.

Equipa Atlas (2008). *Atlas das Aves Nidificantes em Portugal (1999-2005)*. Instituto da Conservação da Natureza e da Biodiversidade, Sociedade Portuguesa para o Estudo das Aves, Parque Natural da Madeira e Secretaria Regional do Ambiente e do Mar. Assírio & Alvim, Lisboa.

Moreira, F. (1999). Relationships between vegetation structure and breeding bird densities in fallow cereal steppes in Castro Verde, Portugal. *Bird Study, 46*, 309-318. doi:10.1080/00063659909461144

Reino, L., Beja, P., Osborne, P.E., Morgado, R., Fabião, A., & Rotenberry, J.T. (2009). Distance to edges, edge contrast and landscape fragmentation: Interactions affecting farmland birds around forest plantations. *Biological Conservation*, *142*, 824–838. doi:10.1016/j.biocon.2008.12.011

Reino, L., Porto, M., Morgado, R., Moreira, F., Fabião, A., Santana, J., Delgado, A., Gordinho, L., Cal, J., & Beja, P. (2010). Effects of changed grazing regimes and habitat fragmentation on Mediterranean grassland birds. *Agriculture, Ecosystems* & *Environment*, *138*, 27-34. doi: 10.1016/j.agee.2010.03.013

Stoate, C., Borralho, R., & Araújo, M. (2000). Factors affecting corn bunting *Miliaria calandra* abundance in a Portuguese agricultural landscape. *Agriculture, Ecosystems* & *Environment*, *77*, 219-226. doi:10.1016/S0167-8809(99)00101-2

Suárez, F., Naveso, M.A., & de Juana, E. (1997). Farming in the drylands of Spain: birds of the pseudosteppes. In Farming and Birds in Europe. In: *The Common Agricultural Policy and its implications for bird conservation* (eds. Pain, D. & Pienkowski, M.W.), Academic Press, San Diego, pp. 297-330.
